# Predicting enhancer transcription and activity from chromatin modifications

**DOI:** 10.1093/nar/gkt826

**Published:** 2013-09-12

**Authors:** Yun Zhu, Lin Sun, Zhao Chen, John W. Whitaker, Tao Wang, Wei Wang

**Affiliations:** ^1^Department of Chemistry and Biochemistry, UCSD, La Jolla 92093, CA, USA and ^2^Department of Cellular and Molecular Medicine, UCSD, La Jolla, CA 92093-0359, USA

## Abstract

Enhancers play a pivotal role in regulating the transcription of distal genes. Although certain chromatin features, such as the histone acetyltransferase P300 and the histone modification H3K4me1, indicate the presence of enhancers, only a fraction of enhancers are functionally active. Individual chromatin marks, such as H3K27ac and H3K27me3, have been identified to distinguish active from inactive enhancers. However, the systematic identification of the most informative single modification, or combination thereof, is still lacking. Furthermore, the discovery of enhancer RNAs (eRNAs) provides an alternative approach to directly predicting enhancer activity. However, it remains challenging to link chromatin modifications to eRNA transcription. Herein, we develop a logistic regression model to unravel the relationship between chromatin modifications and eRNA synthesis. We perform a systematic assessment of 24 chromatin modifications in fetal lung fibroblast and demonstrate that a combination of four modifications is sufficient to accurately predict eRNA transcription. Furthermore, we compare the ability of eRNAs and H3K27ac to discriminate enhancer activity. We demonstrate that eRNA is more indicative of enhancer activity. Finally, we apply our fibroblast trained model to six other cell-types and successfully predict eRNA synthesis. Thus, we demonstrate the learned relationships are general and independent of cell-type. We provided a powerful tool to identify active enhancers and reveal the relationship between chromatin modifications, eRNA production and enhancer activity.

## INTRODUCTION

Distal-acting enhancers are key elements in the regulatory processes that establish cell-type-specific patterns of gene expression. Genome-wide identification of functionally active enhancers is necessary to understand the expression of genes, as well as developmental, and disease-related, processes. Chromatin immunoprecipitation coupled with massively parallel sequencing (ChIP-seq) has allowed the genome-wide mapping enhancers by virtue of their specific chromatin features (1,2). Enhancers are known to be preferentially occupied by sequence-specific DNA-binding proteins and co-activators, such as P300 (3–5). Moreover, enhancers are enriched with H3K4me1 (6–8) and are located in open chromatin regions, thereby displaying DNase-I hypersensitivity (9–12).

Although a large portion of the genome possess enhancer-related chromatin features, only a fraction of enhancers are functionally active. H3K27ac, in combination with H3K4me1, is an important indicator of enhancer activity (13,14). Other marks, such as H3K4me3 and H3K36me3, are also related to enhancer activity (15,16). In contrast, poised enhancers are not functionally active but can be activated during differentiation or in response to external stimuli (13,17). Poised enhancers are marked by H3K27me3 and H3K9me3. During the differentiation of embryonic stem cells (ESC), there is lineage-specific replacement of H3K27me3 with H3K27ac, resulting in the activation of lineage-specific enhancers (16–18).

A more direct indicator of enhancer activity has emerged from a recent genome-wide study that identified many short (<2 kb) non-coding RNAs, which are bi-directionally transcribed from enhancers, and are termed enhancer RNAs (eRNAs) (19,20). The expression of eRNAs from enhancers correlates with the expression of nearby genes and only occurs in the presence of a target promoter. Thus, it suggests that interaction between an active promoter and its regulatory enhancers is necessary for the synthesis of eRNAs (19,21).

Both eRNAs and chromatin marks modifications have been used to identify active enhancers (13,17,20,22); however, their relationship remains largely obscure. It is controversial as to which one of these features is a more robust indicator of enhancer activity. Moreover, it is unclear whether the integration of multiple chromatin modifications can improve the identification of active enhancer.

Herein, we systematically assessed the relationship between chromatin modification, eRNA transcription and enhancer activity. Three questions are specifically addressed: (i) What is the relationship between chromatin modifications and eRNA transcription? (ii) How does this relationship contribute to the identification of active enhancers? (iii) Is this relationship cell-type-specific?

To this end, we integrated ChIP-seq for 24 chromatin modifications and global run-on sequencing (GRO-seq), which provide the eRNA expression levels, in fetal lung fibroblast (IMR90) cells. We developed a logistic regression model that uses chromatin modifications as predictors and GRO-seq levels as response variable. We show that the combination of four modifications can accurately predict eRNA transcription. We further applied this relationship to identify active enhancers. We demonstrated that no single chromatin modification is associated with enhancer activity; instead, eRNA transcription and a combination of chromatin modification can best predict enhancer activity. Finally, we ascertained that this relationship is general, as demonstrated by successfully using a model trained on IMR90 to predict enhancer activity in six different cell-types.

## MATERIALS AND METHODS

### Data

Genome-wide maps of 24 chromatin marks in IMR90 cells were downloaded from the website of the NIH Roadmap Epigenomics Mapping Consortium (http://www.roadmapepigenomics.org, see Supplementary Table S1 for the complete list of 24 marks) (23,24). The IMR90 GRO-seq data were obtained from (25). The downloaded files were mapped reads in BED format. The peaks of the IMR90 P300 ChIP-seq data were called using model-based analysis for ChiP-Seq (MACS) (26). To avoid confounding active transcripts within gene bodies, we focused on intergenic enhancers, which were defined as those outside gene bodies and at least 3 kb from H3K4me3-enriched regions or a known transcription start site (TSS) of a gene (downloaded from the UCSC table browser; http://genome.ucsc.edu/cgi-bin/hgTables?command=start), as was done by Wang *et al.* (20). The ‘Duke Uniq35’ mappability data were downloaded from the UCSC table browser (http://genome.ucsc.edu/cgi-bin/hgTables?command= start). The average mappability for each intergenic enhancer was calculated, and the enhancers with an average mappability of <0.85 were filtered out. For the remaining intergenic enhancers, the read densities within each enhancer region were averaged and transformed to a logarithmic scale, 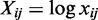 , 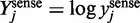, 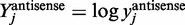, where 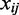 is the average read density for the *i*-th chromatin mark at the *j*-th enhancer and 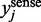 and 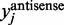 are the sense and anti-sense GRO-seq read densities at the *j*-th enhancer, respectively. The histogram of 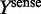 contains two peaks, corresponding to eRNAs with high and low expression (Supplementary Figure S1). K-means (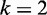) clustering was performed on 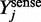 to partition enhancers into GRO-seq^+^ and GRO-seq^−^ on the sense strand. Similarly, another K-means clustering was performed on 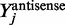 to partition enhancers into GRO-seq^+^ and GRO-seq^−^ on the anti-sense strand. Because eRNAs are bi-directionally transcribed, eRNA^+^ enhancers were defined as those that are GRO-seq^+^ on both the sense and anti-sense strands, whereas eRNA^−^ enhancers were defined as those that are GRO-seq^−^ on both the sense and anti-sense strands. We used eRNA^+^ and eRNA^−^ enhancers as positive and negative examples for the logistic regression model, respectively. We identified a total of 28 959 P300-bound enhancers, of which 14 582 are in intergenic regions. Up to 4117 enhancers whose average mappability values were <0.85 were filtered out, and 4948 eRNA^+^ and 3642 eRNA^−^ enhancers were identified.

### Logistic regression model

Let 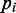 be the probability that the 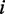 -th enhancer is eRNA^+^ and 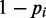 be the probability that it is eRNA^−^. The logistic regression model is

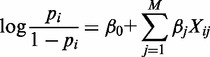

where 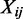 is the 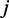 -th histone modification from the 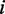-th enhancer, and 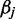 is the regression coefficient that determines the weight of the contribution from the 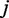 -th histone modification.

### Nested cross-validation

Nested cross-validation includes two nested loops of cross-validations, namely, inner and outer cross-validations (Supplementary Figure S4). Specifically, we first split the data into K folds (K = 10), using K-1 folds as training data and the remaining fold as test data. This approach is the outer cross-validation. Within each loop of outer cross-validation, we performed K-fold inner cross-validation to select top models. The selected top models were then evaluated using the test data in the outer cross-validation. We repeated K loops of outer cross-validation and selected the final top models. As the models selected in the inner loop were not exposed to the test data in the outer loop, nested cross-validation avoids over-fitting when the number of candidate models is large.

### Performance measure

The overall quality of the logistic regression model was measured using the area under the curve (AUC) and Matthew’s correlation coefficient (MCC). The input to the trained classifier is a set of chromatin modifications for an enhancer, whereas the output is the probability that this enhancer produces eRNA. Setting a threshold for this probability produced a particular rate of true and false positives with respect to this threshold. As such, MCC was defined as

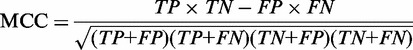

where 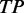 is the number of true positives, 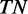 is the number of true negatives, 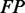 is the number of false positives, and 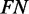 is the number of false negatives. The receiver operating characteristic curve plotted sensitivity as a function of specificity as the threshold varies from 0 to 1. AUC is a quantitative measure of the quality of the classifier. A classifier that correctly identified all eRNA^+^ and eRNA^−^ enhancers would receive an AUC of 1, whereas a random classification would receive an AUC of 0.5.

### Prediction of eRNA expression on H3K4me1^+^me3^−^ enhancers

Peaks of H3K4me1 were called using MACS (26). As we had done for P300-bound enhancers, we focused on intergenic regions by filtering out H3K4me1 peaks within gene bodies or within 3 kb from H3K4me3-enriched regions or from a known TSS of a gene (downloaded from http://genome.ucsc.edu/cgi-bin/hgTables?command=start). As was done for P300-bound enhancers, K-means clustering was performed on both the sense and anti-sense strands, and 6506 eRNA^+^ and 4950 eRNA^−^ enhancers were identified. We applied the model trained on P300-bound enhancers to this set of H3K4me1^+^me3^−^ enhancers.

### Analysis of expression of enhancer-associated genes

IMR90 RNA-seq data were obtained from the NIH Roadmap Epigenomics Mapping Consortium (http://www.roadmapepigenomics.org) (23,24). We used Cufflinks (27) to calculate fragments per kilobase of transcript per million mapped reads based on the RefSeq gene model annotation (downloaded from the UCSC table browser; http://genome.ucsc.edu/cgi-bin/hgTables?command=start). Peaks of H3K27ac ChIP-seq data were called using MACS. Enhancers located within 2 kb of regions enriched for H3K27ac were defined as H3K27ac^+^ enhancers. Other enhancers were defined as H3K27ac^−^ enhancers, as previously reported (17). eRNA^+^ and eRNA^−^ enhancers were identified using K-means clustering on GRO-seq data, as described earlier in the text (see ‘Data’ in ‘Materials and Methods’ section). Predicted eRNA-positive (P-eRNA^+^) and eRNA-negative (P-eRNA^−^) enhancers were derived from the logistic regression model. Each enhancer was assigned to its closest gene based on the distance to a TSS and considering a maximum distance of 100 kb, as described by Rada-Iglesias *et al.* (17).

### Prediction of eRNA expression and enhancer activity in different cell-types

The genome-wide map of the six chromatin modifications (see Supplementary Table S1 for a list of the six marks) and the histone acetyltransferase P300 in the mouse ESC (mESC) cells were obtained from (13) and (28). The chromatin modifications and P300 ChIP-seq data were mapped to the mm9 genome using Bowtie (29). Peaks were called using MACS (26). As with the IMR90 cells, we focused on intergenic enhancers by filtering out P300 peaks within gene bodies or within 3 kb from H3K4me3-enriched regions or a known TSS of a gene (downloaded from http://genome.ucsc.edu/cgi-bin/hgTables?command=start). The mESC GRO-seq data are publicly available in BED format (30). As with the IMR90 cells, K-means clustering was performed on both the sense and anti-sense strands, and 3762 eRNA^+^ and 3607 eRNA^−^ enhancers were identified. In addition, H3K27ac peaks were called using MACS. For purposes of validating the activity of the enhancers, a list of putative enhancers tested for *in vitro* activity in gene reporter assays was taken from (31).

The genome-wide map of chromatin modifications in H1-ESC and the four H1-derived cells [mesendoderm cells (ME), trophoblast-like cells (TBL), mesenchymal stem cells (MSC) and neuronal progenitor cells (NPC)] was downloaded from the website of the NIH Roadmap Epigenomics Mapping Consortium (http://www.roadmapepigenomics.org) (23,24,32). Because we trained our model on IMR90 data, we used only the chromatin marks that overlapped with the 24 IMR90 marks (see Supplementary Table S1 for a complete list of the chromatin marks in the five cell-types). We used P300 ChIP-seq data to identify putative enhancers in the H1 cells. Because the P300 ChIP-seq data are not available for the four H1-derived cells, the enhancers in these four cell lines were defined as the peaks of the H3K4me1-enriched regions and were required to be located at least 3 kb away from the H3K4me3-enriched regions or a known TSS. We again filtered out enhancers whose mappability values were <0.8 and identified a total of 14 724 enhancers in H1 cells, 37 215 enhancers in ME cells, 31 803 enhancers in TBL cells, 25 555 enhancers in MSC cells and 25 473 enhancers in NPC cells. We then applied our logistic regression model trained on IMR90 data to the five cell-types to predict eRNA^+^ and eRNA^−^ enhancers. As with the IMR90 cells, we used MACS to call H3K27ac peaks in these five cell-types.

### Luciferase reporter assay

Fourteen enhancers comprising four subgroups (eRNA^+^K27ac^+^, eRNA^+^K27ac^−^, eRNA^−^K27ac^+^ and eRNA^−^ K27ac^−^) were amplified from the genomic DNA and cloned into the enhancer site of pGL3-promoter vectors, and the inserted sequences were verified by DNA sequencing. IMR90 cells were maintained following the protocol of the American Type Culture Collection. Briefly, cells were grown in MEM supplemented with 10% fetal bovine serum (FBS), 1% pen/strep, 1% NEAA and 1% sodium pyruvate and split 1:3 every 3 days. Cells were seeded into 24-well plates 1 day before transfections. The cells were transfected with 500 ng of a pGL3 plasmid and 50 ng pRL-CMV, washed with PBS 24 h after transfection and then lysed for 20 min. Luciferase activity was measured using the dual luciferase assay (Promega) using a Veritas microplate luminometer (Turner BioSystems), normalized to Renilla luciferase activity and then divided by the values for a pGL3 minimal promoter empty vector control. All assays were performed in triplicate.

## RESULTS

### Deciphering the relationship between chromatin modifications and enhancer transcription

**Figure 1.**
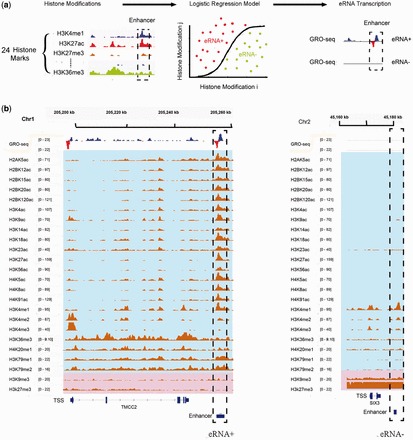
Methodological overview. (**a**) The levels of 24 chromatin marks at each P300-bound enhancer were used as inputs to optimize a logistic regression model that best separates eRNA^+^ (positive) and eRNA^−^ (negative) training samples. (**b**) Sample profiles of the 24 histone modifications for the eRNA^+^ (left) and eRNA^−^ (right) enhancers. Both enhancers are enriched with H3K4me1 but depleted of H3K4me3. The left enhancer is actively producing bi-directional eRNA transcripts and is enriched with histone acetylations and H3K79me1 but depleted of H3K27me3 and H3K9me3. No eRNA transcripts were detected on the right enhancer locus, which is enriched in H3K27me3 and H3K9me3 but depleted of histone acetylations and H3K79me1.

To understand the relationship between chromatin modifications and eRNA transcription, we collected ChIP-seq for 24 chromatin modifications in human IMR90 cells (23,24) and GRO-seq levels at enhancers (25) (Figure 1b, Supplementary Table S1, see also ‘Materials and Methods’ section). Binding of the transcriptional coactivator P300 often marks distal enhancers (5,6,33). Using P300 ChIP-seq data, we identified P300-bound enhancers in IMR90 cells and further used GRO-seq data to classify them into eRNA^+^ and eRNA^−^ enhancers.

We developed a logistic regression model to study the relationship between chromatin modifications and enhancer transcription. In the training phase, we used eRNA^+^ and eRNA^−^ enhancers as positive and negative examples, respectively, and a set of regression coefficients was estimated. In the test phase, the estimated regression coefficients were used to distinguish differentially expressed eRNAs in a test data set (Figure 1a, see also ‘Materials and Methods’ section). To test our models performance, we used 10-fold cross-validation. This maximizes the use of the data set and ensures the learned relationships are general and not limited to a subset of enhancers. Performance was evaluated using the AUC of the receiver operating characteristic and MCC for the model (see ‘Materials and Methods’ section).

**Figure 2.**
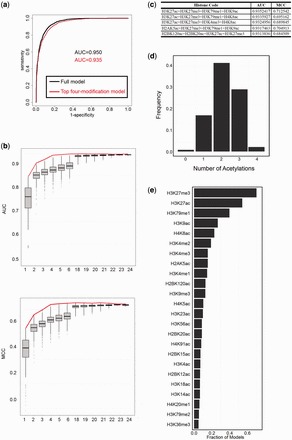
Chromatin modifications predict eRNA synthesis. (**a**) AUCs obtained using the full and top four-modification models. (**b**) Distribution of AUCs and MCCs using all *m*-modification models with *m* taken from 1 to 6 and from 18 to 24. Because the number of combinations of 7–17 variables was too high, they were omitted. The red curves represent AUC and MCC from the top *m*-modification model, respectively. (**c**) Five of the top four-modification models with their AUCs and MCCs. (**d**) The number of acetylations in the best-scored four-modification models. The best-scored four-modification models were the ones with at least 95% of the AUCs and MCCs obtained with the full model. (**e**) The frequency of appearance of the 24 chromatin marks in the best-scored four-modification models.

We started with the full model, which includes all 24 chromatin marks. The full model has an AUC of 0.95 and MCC of 0.75. It clearly demonstrates that chromatin modifications can discriminate enhancers according to eRNA expression levels (Figure 2a). We next asked whether all of the modifications are required in the logistic regression model. To this end, we then chose 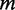 of the 24 modifications (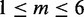
_,_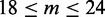 ) and constructed all possible 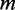 -modification models. To avoid overfitting a large number of models, we used nested cross-validation (see ‘Materials and Methods’ section). The top one-modification model (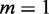) is the one that uses H3K27ac. It has an AUC of 0.84 and MCC of 0.54 (Supplementary Figure S2a and b). As one increases *m*, the AUCs and MCCs of the best-performing *m*-modification model increase (red curve in Figure 2b, *P* = 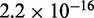

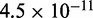
 and 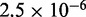 for *m* = 1, 2, and 3, respectively, Wilcoxon test). However, the improvement is insignificant when more than four modifications are used (*P* = 0.237 for *m* = 4, Wilcoxon test). Taken together, these findings suggest that the incorporation of multiple chromatin marks performs better than using only H3K27ac but that four modifications are sufficient.

To identify a subset of modifications with high predictive power, we selected the top 5% of models in terms of AUC and MCC (see Figure 2c and Supplementary Table S2 for a complete list). The best-performing model is H3K27ac+H3K27me3+H3K79me1+H3K9ac, including two acetylations and two methylations. Of the selected 432 models, 99.1% have at least one histone acetylation (Figure 2d). Approximately half of the models have two acetylations; the other half has one or three acetylations. Only 2.1% have four acetylations. The best model with four acetylations has an AUC of 0.916 and MCC of 0.66, which are lower than those of the best-performing model (AUC = 0.935 and MCC = 0.712). These findings imply that acetylations are important but that incorporation of chromatin modifications other than histone acetylations provides complementary information.

Of the 432 selected models, H3K27ac and H3K27me3 were the top two overrepresented marks (Figure 2e, *P* = 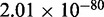 and 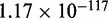, respectively, hypergeometric test). H3K27ac has a positive regression coefficient, indicating that it is positively correlated with eRNA expression. In contrast, H3K27me3 has a negative regression coefficient, indicating a negative correlation with eRNA expression. These observations are not unexpected as these two marks are known to be associated with enhancer activity, and it suggests that eRNA expression is also related to enhancer activity (see later in the text).

In addition to H3K27ac and H3K27me3, our analysis identified a set of novel chromatin marks that are highly predictive of eRNA production. We found that 41% of the selected models included H3K79me1 (Figure 2e, *P* = 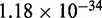, hypergeometric test). This chromatin modification is known to be enriched at the promoters of active genes (34). Our results suggest that this modification is also associated with eRNA production. Furthermore, two other acetylation modifications, H3K9ac and H4K8ac, are also significantly overrepresented in the selected models (*P* = 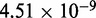 and 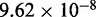, respectively, hypergeometric test). Moreover, all three of these modifications have positive regression coefficients in the full model (Supplementary Figure S2c), indicating their positive contribution to eRNA expression. These findings imply that H3K79me1, H3K8ac and H3K9ac are novel chromatin signatures that are associated with active enhancers (see later in the text).

Notably, the most frequently observed chromatin marks do not necessarily have the largest individual predictive power (Supplementary Figure S2a and b). For instance, H3K27me3 is the most frequently selected of the 24 chromatin modifications, although its individual predictive power is not the highest. When H3K27me3 is combined with other modifications, the combined model achieves greater accuracy than any single modification in isolation, which indicates that integration of multiple chromatin marks improves prediction accuracy.

To test whether the derived model and chromatin features are general to a broader set of enhancers, not necessarily only those bound by P300, we applied the best-performing four-modification model to a broader set of enhancers that are enriched for H3K4me1 and depleted in H3K4me3 (6–8) (see ‘Materials and Methods’ section). The model obtained an AUC of 0.93, close to the value obtained when the model was both trained and tested on P300-bound enhancers (Supplementary Figure S5a). Moreover, only 62% of the predicted eRNA^+^ enhancers were bound by P300. These findings indicate that the chromatin features associated with eRNA production are common and not limited to P300-bound enhancers.

### The discriminative model distinguishes active from inactive enhancers

**Figure 3.**
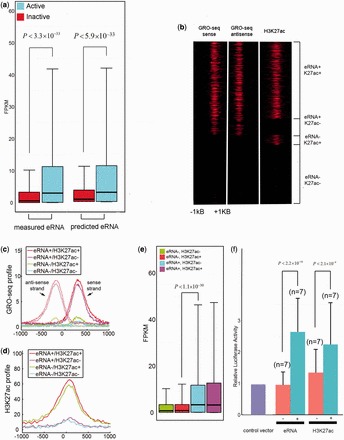
eRNA transcription is a robust indicator of active enhancers. (**a**) Expression levels of genes associated with active and inactive enhancers identified by three different marks: measured eRNAs, predicted eRNAs and H3K27ac. (**b**) Heatmaps of GRO-seq and H3K27ac signals for four classes of enhancers: eRNA^+^K27ac^+^, eRNA^+^K27ac^−^, eRNA^-^K27ac^+^ and eRNA^−^K27ac^−^. The enhancer windows were centered at p300-binding peaks. (**c**) Average profiles of GRO-seq signals for the four classes of enhancers in (b). (**d**) Average profiles of H3K27ac signals for the four classes of enhancers in (b). (**e**) Expression levels of the genes associated with the four classes of enhancers in (b). (**f**) Luciferase assay testing the enhancer activity for the eRNA^+^, eRNA^−^, H3K27ac^+^ and H3K27ac^−^ enhancers.

Given the good agreement between the measured and predicted eRNA expression levels, we proceeded to analyze the relationship between these levels and enhancer activity. We first used measured eRNAs to separate enhancers into eRNA^+^ and eRNA^−^ enhancers. Then we compared the expression levels of each enhancers nearest gene. Our results show that eRNA^+^-enhancer-associated gene expression is significantly higher than eRNA^−^-enhancer-associated gene expression (Figure 3a, *P* = 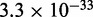, Wilcoxon test).

We next applied the best-performing four-modification model (H3K27me3+H3K27ac+H3K79me1+H3K9ac) to classify enhancers into predicted eRNA-positive (P-eRNA^+^) and eRNA-negative (P-eRNA^−^) elements. The P-eRNA^+^ enhancers have significantly higher levels of associated gene expression than the P-eRNA^−^ enhancers. More importantly, the significance level of gene expression is comparable with the significance using measured eRNA levels (Figure 3a, *P* = 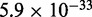, Wilcoxon test). These results imply that both measured and predicted eRNAs distinguish active from inactive enhancers.

As both eRNA levels and H3K27ac are associated with enhancer activity (13,20,22), it is interesting to ask which feature is a more robust indicator of enhancer activity. We separated enhancers into H3K27ac^+^ and H3K27ac^−^ categories and found that the gene expression levels of H3K27ac^+^ enhancers are higher than those of H3K27ac^−^ enhancers. However, the significance level (*P* = 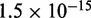, Wilcoxon test) is much lower than the significance using either measured or predicted eRNA transcription.

We further divided enhancers based on their predicted eRNA expression into four subgroups: p-eRNA^+^K27ac^+^, p-eRNA^+^K27ac^−^, p-eRNA^-^K27ac^+^ and p-eRNA^-^K27ac^−^ (Figure 3b). The GRO-seq and H3K27ac enrichment profiles of the four subgroups are shown in Figure 3c and d. Strikingly, the expression levels of p-eRNA^+^K27ac^−^ enhancer-associated gene are significantly higher than those of p-eRNA^-^K27ac^+^ enhancer-associated genes (Figure 3e, *P* = 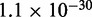, Wilcoxon test).

To test whether the observations are limited to P300-bound enhancers, we applied the best-performing four-modification model to H3K4me1^+^me3^−^ enhancers and repeated the aforementioned analyses. The results for H3K4me1^+^me3^−^ enhancers are similar to the results for P300-bound enhancers. Briefly, both measured and predicted eRNAs separate enhancers, in terms of their associated gene expression (Supplementary Figure S5b, *P* = 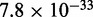 for measured eRNAs and 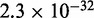 for predicted eRNAs, Wilcoxon test). Moreover, p-eRNA^+^K27ac^−^ enhancer-associated genes show significantly higher expression levels than p-eRNA^-^K27ac^+^ enhancer-associated genes (Supplementary Figure S5c, *P* = 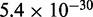, Wilcoxon test). This finding indicates that the observations are general and not limited to P300-bound enhancers.

To experimentally verify the relationship between eRNA transcription, H3K27ac enrichment and enhancer activity, we randomly selected 14 enhancers from the four subgroups (Supplementary Table S3) and investigated their activity (Figure 3f and Supplementary Figure S3). Both eRNA production and H3K27ac enrichment are positively correlated with luciferase activity. However, the difference in luciferase activity is much more significant between the p-eRNA^+^ and p-eRNA^−^ enhancers (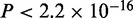) than between the H3K27ac^+^ and H3K27ac^−^ enhancers (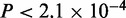). The accuracy improved from 5/9 to 7/9 with the use of predicted eRNA production instead of H3K27ac to identify active enhancers. Notably, all three p-eRNA^+^K27ac^−^ enhancers were positive in the luciferase reporter assay. In comparison, only one of three p-eRNA^−^K27ac^+^ enhancers was positive.

Taken together, these findings suggest that eRNA production is a more reliable indicator of enhancer activity compared with H3K27ac. Furthermore, H3K27ac is not the only chromatin modification that is associated with enhancer activity. Our enhancer activity prediction model, which incorporates four chromatin modifications, provides a strong performance improvement over any single modification. The model is useful because it provides a method for accurately identifying active enhancers when GRO-seq data are not available or do not have sufficient sequence depth to identify all active enhancers.

### The discriminative model is cell-type independent

To investigate the generality of our logistic regression model, we applied it to different cell-types. We used six publicly available chromatin modifications (H3K4me1, H3K4me3, H3K27me3, H3K27ac, H3K36me3 and H3K9me3), GRO-seq and P300 ChIP-seq in mESC (see ‘Materials and Methods’ section).

**Figure 4.**
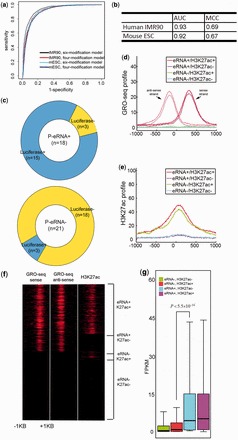

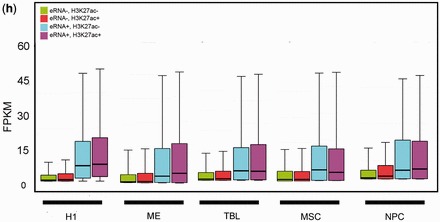
Application of the logistic regression model across six cell-types. (**a**) AUCs of the top six- and four-modification models tested on the IMR90 and mESC cells. All models were trained on IMR90 cells. (**b**) Performance comparison of the top four-modification models, which were trained and tested on the same (IMR90) and different (trained on IMR90 but tested on mESC) cell-types. (**c**) Of the 18 predicted eRNA^+^ (P-eRNA^+^) enhancers, 15 were positive in luciferase reporter assays. Of the 21 predicted eRNA^−^ (P-eRNA^−^) enhancers, only three were positive in luciferase reporter assays. (**d**) Average profiles of GRO-seq signals for the four classes of enhancers in mESC cells: eRNA^+^K27ac^+^, eRNA^+^K27ac^−^, eRNA^−^K27ac^+^ and eRNA^−^K27ac^−^. The enhancer windows were centered at p300-binding peaks. (**e**) Average profiles of H3K27ac for the four classes of enhancers in mESC cells. (**f**) Heatmaps of GRO-seq and H3K27ac signals for the four classes of enhancers in mESC cells. (**g**) Expression levels of genes associated with the four classes of enhancers in mESC cells. (**h**) Expression levels of genes associated with the four classes of enhancers in H1, ME, TBL, MSC and NPC cells.

The six chromatin modifications in mESCs were also available in IMR90. Thus, we trained the model using these six modifications in IMR90 cells and evaluated it in mESC. This model has an AUC of 0.93 and MCC of 0.69, which are comparable with the AUC and MCC values (AUC = 0.93 and MCC = 0.70) obtained when the model is trained and tested using the same six chromatin modifications in IMR90 cells (Figure 4a and b). The best-performing four-modification model is H3K27ac+H3K27me3+H3K4me3+H3K9me3. This model has an AUC of 0.92 and MCC of 0.67, values that are comparable with those obtained for the model when it was both trained and tested using IMR90 cells (AUC = 0.93 and MCC = 0.69) (Figure 4a and b). These results indicate that our logistic regression model is cell-type-independent and that the relationship between chromatin modifications and eRNA transcription revealed by our model is general.

We next examined whether eRNA transcription remains more indicative of enhancer activity than H3K27ac in mESC cells. As with human IMR90 cells, we classified mESC enhancers into four subgroups based on their levels of predicted eRNAs and H3K27ac. P-eRNA^+^K27ac^+^ and p-eRNA^+^K27ac^−^ enhancers have high-level bi-modal GRO-seq signals (Figure 4d), whereas p-eRNA^+^K27ac^+^ and p-eRNA^-^K27ac^+^ enhancers are enriched for H3K27ac (Figure 4d, e and f). Consistent with the observations in IMR90 cells, p-eRNA^+^ enhancer-associated genes, regardless of their H3K27ac levels, have significantly higher expression levels than p-eRNA^−^ enhancer-associated genes. Furthermore, the difference in gene expression level between p-eRNA^+^K27ac^−^ and p-eRNA^-^K27ac^+^ enhancers is significant (*P* = 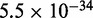, Wilcoxon test).

To further verify the relationship between enhancer activity and eRNA levels, we collected 67 mESC enhancers, whose activities were previously studied using luciferase reporter assays (16,31). This set included 39 intergenic enhancers. The top four-modification model identified 18 active enhancers, of which 15 were positive in the luciferase reporter assay (83.3%, compared with 67% if using H3K27ac alone, Figure 4c). We also identified 21 inactive enhancers, of which only three were positive (14.3%, compared with 20% if using H3K27ac alone, Figure 4c). These results further confirm that eRNA production is predictive of enhancer activity and that our logistic regression model has a higher predictive power than H3K27ac alone.

In addition to mESCs, we also applied our logistic regression model to H1 cells and four H1-derived cells (32): MEs, TBLs, MSCs and NPCs. Using the available chromatin marks in these five cell-types, we selected the top four marks whose combination exhibited the highest performance and used our logistic regression model to predict eRNA^+^ and eRNA^−^ enhancers (see ‘Materials and Methods’ section and Supplementary Table S1). In all five cell-types, p-eRNA^+^K27ac^−^ enhancers were associated with significantly higher levels of gene expression than the p-eRNA^-^K27ac^+^ enhancers (Figure 4g and h). These results indicate that our logistic regression model using four chromatin marks has greater discriminative power in identifying active enhancers than H3K27ac alone, irrespective of cell-type.

## DISCUSSION

Herein, we uncover the relationship between chromatin modifications, eRNA synthesis and enhancer activity. Several recent studies have also explored the relationship between chromatin modifications and enhancer activity (13,16,17); however, our study expands on these in the following ways. First, to the best of our knowledge, this study is the first to unravel the relationship between chromatin modifications and eRNA synthesis. Thus, one can infer eRNA expression levels and more robustly identify active enhancers in a cell-type without eRNA data. Currently, GRO-seq results are available for only a limited number of cell-types, and the sequencing depth may be insufficient to identify all active enhancers. Thus, our study provides powerful methodology for predicting enhancer activity from combinations of histone modifications. As histone modification ChIP-seq experiments often have stronger signals than GRO-seq, the most reliable set of eRNA producing active enhancers can be used to train the model and make predictions in other cell-types.

Second, instead of linking enhancer activity to single chromatin marks, such as H3K27ac and H3K27me3, our study systematically examined combinations of 24 chromatin marks and deciphered a combinatorial ‘histone code’ for enhancer activity. We have demonstrated that only a few chromatin modifications are necessary to accurately predict enhancer activity. This can be understood if the chromatin modifications are classified into different groups, i.e. associated, or unassociated, with eRNA production. Alternatively, these modifications could be involved in different steps of eRNA transcription. Chromatin modifications in the same group are redundant, and our logistic regression model selects the most representative ones from each group. The 24 modifications can be classified into three groups: (i) Acetylations. In corroboration of previous reports that histone acetylations are redundant (2), our logistic regression model selects H3K27ac, H4K8ac and H3K9ac of 15 histone acetylations as the most informative marks with respect to eRNA production. (ii) The repressive modification H3K27me3. This modification is deposited by the polycomb complex PRC1/PRC2 (35–37) and is enriched at inactive enhancers. (iii) H3K79me1 and H3K36me3. These two modifications were previously reported to be associated with PolII elongation within active gene bodies (1,28,38). Interestingly, both PolII and H3K36me3 have also been detected at active enhancer regions (16,18). Therefore, H3K36me3 and H3K79me1 are likely to correlate with eRNA transcription, which is consistent with our observations.

Third, we compared eRNA production and H3K27ac ability to predict enhancer activity and demonstrated that eRNA transcription is more robust. Although the mechanism of eRNA transcription is unclear, this process may involve multiple chromatin modifications. Although H3K27ac might be one of these modifications, it is not the only chromatin mark involved. Thus, the inclusion of multiple chromatin modifications as a ‘histone code’ improves the accuracy of prediction of eRNA transcription. A previous study showed that eRNA transcription depends on the presence of an intact target promoter (19). Thus, it suggests that eRNA synthesis provides direct, and functional, evidence of an enhancers activity. Therefore, the incorporation of multiple chromatin modifications better compartmentalizes enhancers based on their activity.

We trained our model on P300-bound enhancers because this approach allowed us to perform an unbiased analysis on model/chromatin feature selection. However, our analysis is not limited to P300-bound enhancers. The selected chromatin features are general and common to a broader set of enhancers. As described, we applied our model to H3K4me1^+^me3^−^ enhancers. Our model predicted a set of active enhancers, only 62% of which overlapped with P300 binding sites. Furthermore, we applied the top four-modification model to H3K4me1^+^me3^−^ enhancers and found that the expression levels of P-eRNA^+^K27ac^−^ enhancer-associated genes are significantly higher than those of P-eRNA^-^K27ac^+^ enhancer-associated genes. This observation is consistent with the results observed for P300-bound enhancers and thus serves as additional evidence that eRNA expression is a more robust indicator of enhancer activity compared with H3K27ac. Taken together, these findings indicate that the model trained on P300-bound enhancers is not limited to P300-bound enhancers.

## ACKNOWLEDGEMENT

Lin Sun and Zhao Chen contributed equally to this paper.

## SUPPLEMENTARY DATA

Supplementary Data are available at NAR Online.

## FUNDING

Funding for open access: National Institutes of Health [R01GM096194 to W.W. and U01ES017166 to W.W.] (in part).

*Conflict of interest statement*. None declared.

## Supplementary Material

Supplementary Data
