# Supplementary material for: Predicting enhancer transcription and activity from chromatin modifications
Source: Nucleic Acids Res. 2013 Sep 12;41(22):10032–43. doi: 10.1093/nar/gkt826 (PMC3905895; doi:10.1093/nar/gkt826)
Supplement: Supplementary Data [file supp_41_22_10032__index.html]

Predicting enhancer transcription and activity from chromatin modifications — Predicting enhancer transcription and activity from chromatin modifications — Supplementary Data 

# Predicting enhancer transcription and activity from chromatin modifications

## Supplementary Data

files

**Files in this Data Supplement:**

- Supplementary Data - pdf file
