# Supplementary material for: Predicting enhancer transcription and activity from chromatin modifications
Source: Nucleic Acids Res. 2013 Sep 12;41(22):10032–43. doi: 10.1093/nar/gkt826 (PMC3905895; doi:10.1093/nar/gkt826)
Supplement: Supplementary Data [file supp_gkt826_nar-01649-m-2013-File007.pdf]

Supplemental Figure Legends

Supplemental Figure 1: Histograms of the sense (a) and antisense (b) GRO-Seq data. On both strands, the histograms have a bi-modal distribution. The left and right modes correspond to enhancers with weakly and highly expressed eRNAs, respectively.

Supplemental Figure 2: (a) AUCs obtained using single chromatin modifications. (b) MCCs obtained using single chromatin modifications.

Supplemental Figure 3: Validation of enhancer activity using luciferase reporter assays. Results of triplicate luciferase assays on four subgroups of enhancers: $eRNA^{+}K27ac^{+}$, $eRNA^{+}K27ac^{-}$, $eRNA^{-}K27ac^{+}$, and $eRNA^{-}K27ac^{-}$. Relative enhancer activity is indicated as the fold enrichment over the pGL3 promoter vector.

Supplemental Figure 4: Scheme of nested cross validation, showing that model selection is within an inner cross-validation and therefore selected models do not see the test samples in the outer cross validation.

Supplemental Figure 5: Application of the logistic regression model trained on P300-bound enhancers to $H3K4me1^{+}me3^{-}$ enhancers. (a) AUCs obtained using the full and top four-modification models. (b) Expression levels of genes associated with active and inactive enhancers identified by three different marks: measured eRNAs, predicted eRNAs, and H3K27ac. (c) Expression levels of the genes associated with the four classes of enhancers: $eRNA^{+}K27ac^{+}$, $eRNA^{+}K27ac^{-}$, $eRNA^{-}K27ac^{+}$, and $eRNA^{-}K27ac^{-}$.

Supplemental Figure 1

a b

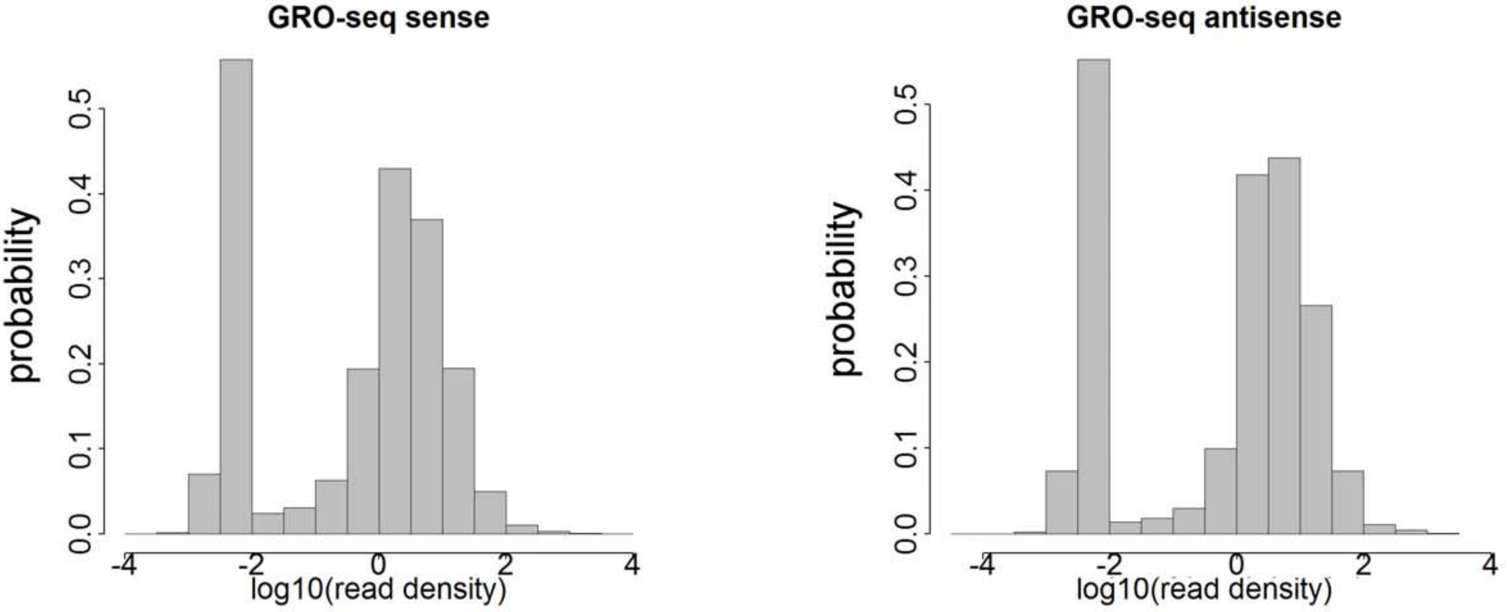

Supplemental Figure 2

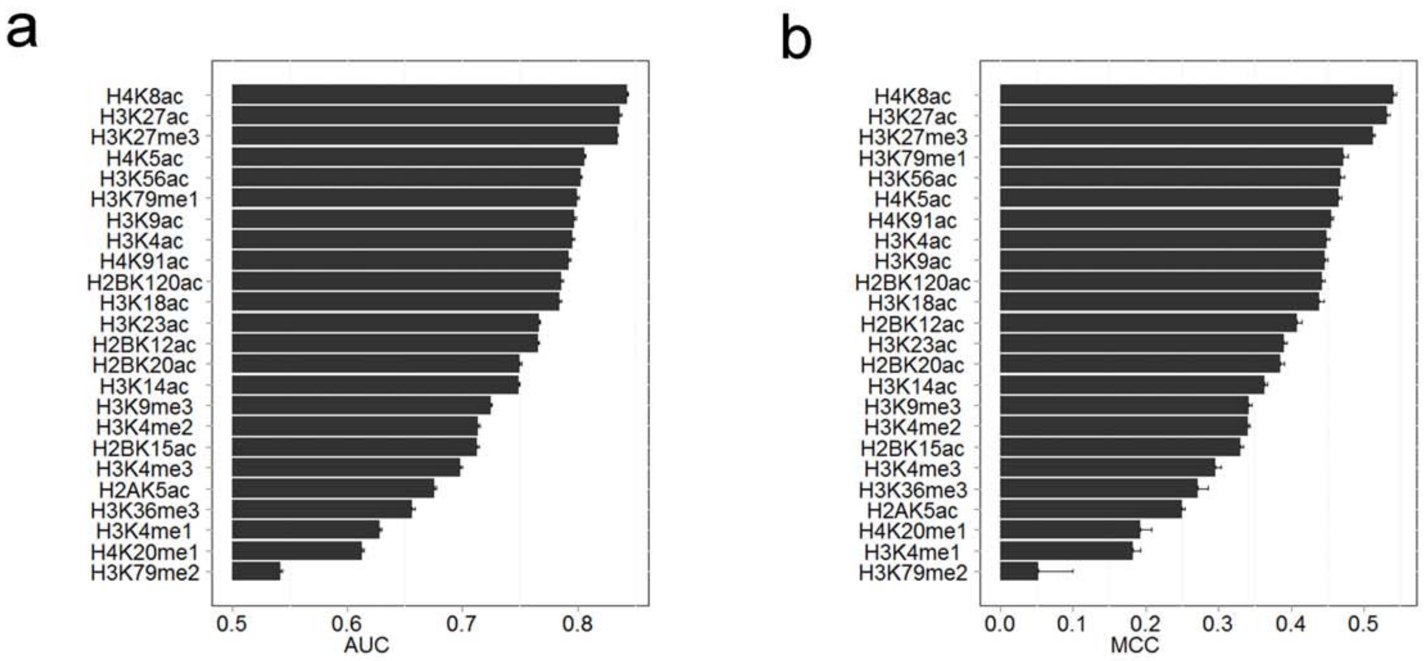

Supplemental Figure 3

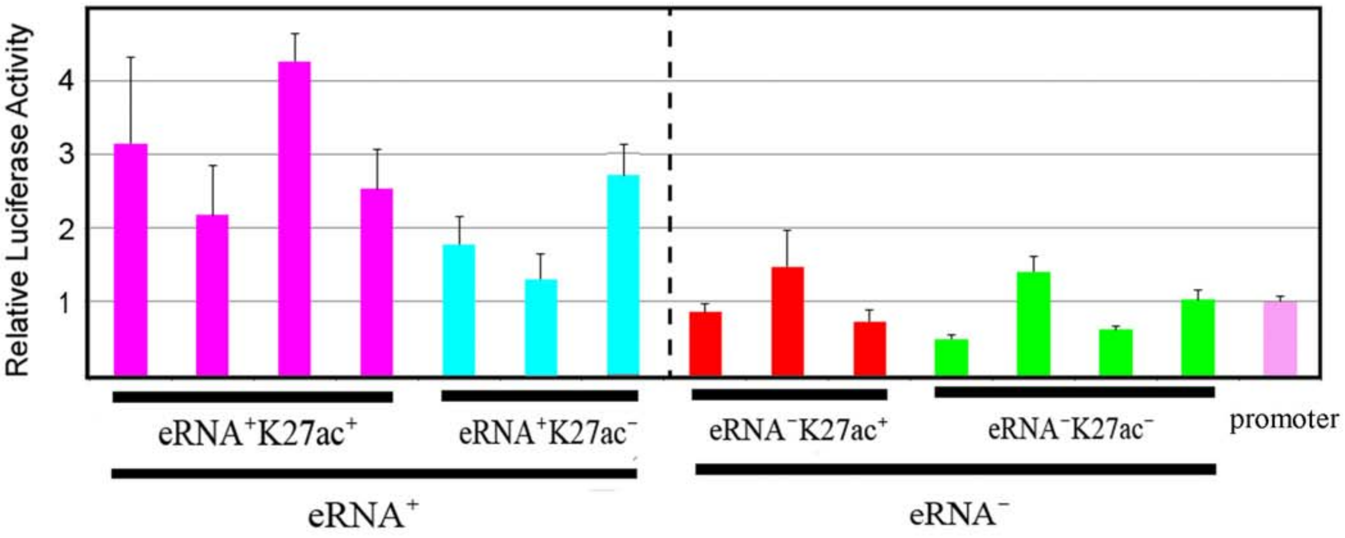

Supplemental Figure 4

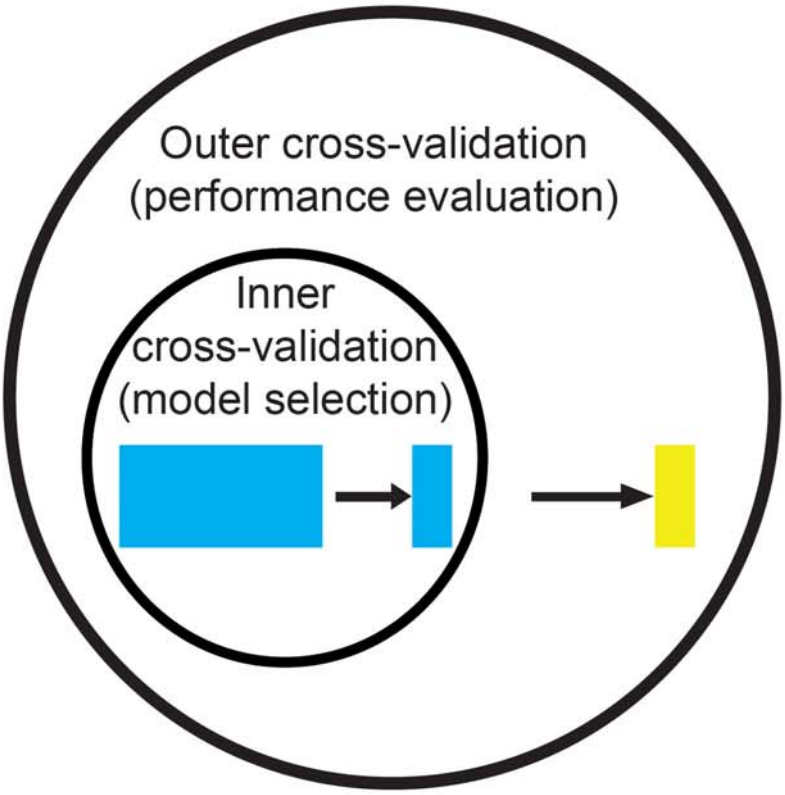

Supplemental Figure 5

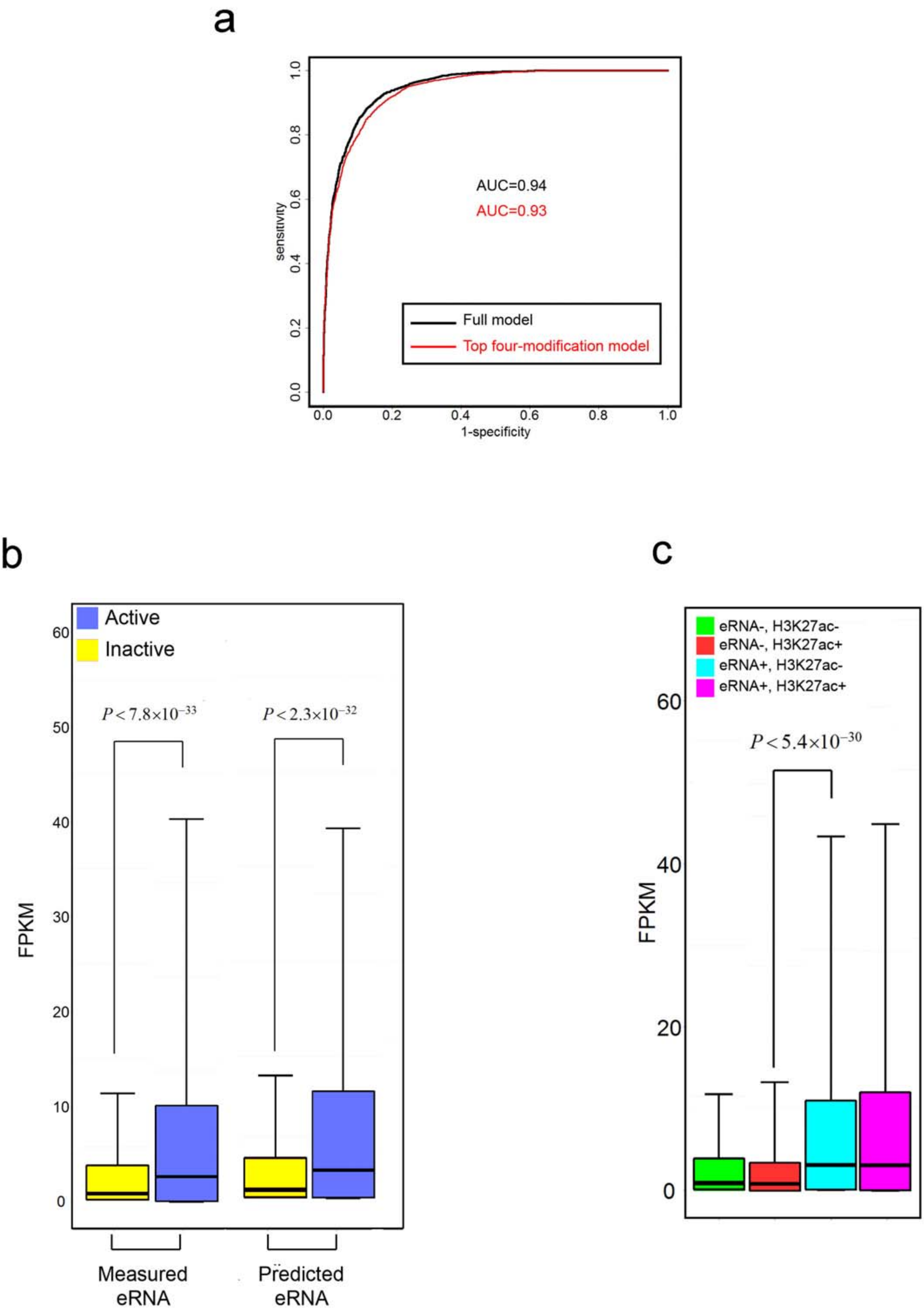

Table S1: Cell lines and chromatin marks involved in the current study

Cell line	Number of chromatin marks	List of chromatin marks	Top four-modification model
IMR90 Human fetal lung fibroblast cells	24	H2AK5ac, H2BK120ac, H2BK12ac, H2BK15ac, H2BK20ac, H3K14ac, H3K18ac, H3K23ac, H3K27ac, H3K27me3, H3K36me3, H3K4ac, H3K4me1, H3K4me2, H3K4me3, H3K56ac, H3K79me1, H3K79me2, H3K9ac, H3K9me3, H4K20me1, H4K5ac, H4K8ac, H4K91ac	H3K27me3+H3K27ac +H3K79me1+H3K9ac
mESC mouse embryonic stem cell	6	H3K4me1, H3K4me3, H3K27ac, H3K27me3, H3K36me3, H3K9me3	H3K27ac+H3K27me3 +H3K4me3+H3K9me3
H1 Human embryonic stem cells, line H1	22	H2AK5ac, H2BK120ac, H2BK12ac, H2BK15ac, H2BK20ac, H3K18ac, H3K23ac, H3K27ac, H3K27me3, H3K36me3, H3K4ac, H3K4me1, H3K4me2, H3K4me3, H3K56ac, H3K79me1, H3K79me2, H3K9ac, H3K9me3, H4K20me1, H4K5ac, H4K91ac	H3K27me3+H3K27ac +H3K79me1+H3K9ac
ME H1 BMP4 derived mesendoderm cultured cells	12	H2AK5ac, H2BK120ac, H3K18ac, H3K23ac, H3K27ac, H3K27me3, H3K36me3, H3K4me1, H3K4me3, H3K79me1, H3K79me2, H3K9ac	H3K27me3+H3K27ac +H3K79me1+H3K9ac
TB H1 BMP4 derived trophoblast cultured cells	17	H2AK5ac, H2BK120ac, H2BK12ac, H3K14ac, H3K18ac, H3K27ac, H3K27me3, H3K36me3, H3K4ac, H3K4me1, H3K4me3, H3K79me1, H3K79me2, H3K9ac, H3K9me3, H4K8ac, H4K91ac	H3K27me3+H3K27ac +H3K79me1+H3K9ac
MSC H1 derived mesenchymal stem cells	18	H2AK5ac, H2BK120ac, H2BK12ac, H3K14ac, H3K18ac, H3K23ac, H3K27ac, H3K27me3, H3K36me3, H3K4ac, H3K4me1, H3K4me2, H3K4me3, H3K79me1, H3K9ac, H3K9me3, H4K8ac, H4K91ac	H3K27me3+H3K27ac +H3K79me1+H3K9ac
NPC H1 derived neuronal progenitor cultured cells	16	H2BK12ac, H3K14ac, H3K18ac, H3K23ac, H3K27ac, H3K27me3, H3K36me3, H3K4ac, H3K4me1, H3K4me2, H3K4me3, H3K79me1, H3K9ac, H3K9me3, H4K8ac, H4K91ac	H3K27me3+H3K27ac +H3K79me1+H3K9ac

Table S2: List of the top 432 four-modification models and their AUCs anc MCCs

Histone Code	AUC	MCC
H3K27ac+H3K27me3+H3K79me1+H3K9ac	0.935242	0.712542
H3K27ac+H3K27me3+H3K79me1+H4K8ac	0.933593	0.695162
H3K27ac+H3K27me3+H3K4me3+H4K8ac	0.932496	0.689845
H2AK5ac+H3K27me3+H3K79me1+H3K9ac	0.931746	0.704913
H2BK120ac+H2BK20ac+H3K27ac+H3K27me3	0.931384	0.684509
H3K18ac+H3K27me3+H3K79me1+H4K8ac	0.931166	0.698695
H2BK15ac+H3K27me3+H3K79me1+H4K8ac	0.931038	0.698581
H3K23ac+H3K27ac+H3K27me3+H3K4me3	0.930882	0.707214
H3K27me3+H3K4me2+H3K79me1+H3K9ac	0.930781	0.7049
H2BK120ac+H3K27ac+H3K27me3+H3K79me1	0.930752	0.70518
H3K27ac+H3K27me3+H3K4me3+H3K9ac	0.930288	0.696054
H3K27ac+H3K27me3+H3K4me3+H4K91ac	0.930216	0.696424
H3K27ac+H3K27me3+H3K4me3+H3K79me1	0.929953	0.681604
H3K27ac+H3K27me3+H3K4me2+H3K9ac	0.929931	0.699718
H2BK120ac+H2BK20ac+H3K27me3+H3K9ac	0.929917	0.677994
H3K27ac+H3K27me3+H3K4me2+H3K79me1	0.929868	0.697372
H2AK5ac+H3K27ac+H3K4me3+H3K9ac	0.929841	0.678152
H2AK5ac+H3K27ac+H3K27me3+H3K9ac	0.929825	0.704078
H3K14ac+H3K27ac+H3K27me3+H3K4me3	0.929761	0.700875
H3K27me3+H3K4ac+H3K79me1+H4K8ac	0.929729	0.69136
H3K27ac+H3K27me3+H3K4me3+H3K9me3	0.929728	0.693474
H3K27ac+H3K27me3+H3K79me1+H4K91ac	0.929706	0.690478
H3K27ac+H3K27me3+H3K4me3+H3K56ac	0.92966	0.692095
H2BK20ac+H3K27ac+H3K27me3+H3K9ac	0.929651	0.69713
H3K27me3+H3K79me1+H3K9ac+H4K8ac	0.929544	0.687543
H2BK15ac+H3K27ac+H3K9ac+H3K9me3	0.929488	0.688676
H3K27ac+H3K4me1+H3K4me2+H3K9ac	0.929486	0.680501
H2AK5ac+H3K27ac+H3K79me2+H3K9ac	0.92948	0.690429
H2AK5ac+H3K27me3+H3K56ac+H3K9ac	0.92946	0.695544
H2BK120ac+H3K27me3+H3K79me1+H4K8ac	0.929316	0.685618
H2BK15ac+H3K27ac+H3K27me3+H3K9ac	0.929293	0.689998
H3K23ac+H3K27ac+H3K27me3+H3K79me1	0.929275	0.698931
H3K27ac+H3K27me3+H3K36me3+H4K8ac	0.929271	0.697761
H3K27ac+H3K27me3+H3K4me2+H4K91ac	0.929242	0.698131
H3K27ac+H3K4me1+H3K56ac+H3K9ac	0.929207	0.6977
H3K27ac+H3K27me3+H3K4me2+H4K8ac	0.929038	0.697798
H2BK12ac+H3K27ac+H3K27me3+H3K4me3	0.928938	0.694857
H2BK15ac+H3K27ac+H3K4me1+H3K9ac	0.928912	0.702584
H2BK15ac+H3K27me3+H3K79me1+H3K9ac	0.928852	0.69272
H3K27me3+H3K56ac+H3K79me1+H4K8ac	0.928817	0.695857
H2AK5ac+H3K27ac+H3K36me3+H3K9ac	0.928816	0.687835
H3K23ac+H3K27me3+H3K79me1+H4K8ac	0.928739	0.693963
H2AK5ac+H3K27ac+H3K79me1+H3K9ac	0.928647	0.686124
H2BK20ac+H3K27ac+H3K27me3+H4K8ac	0.928579	0.679028
H3K27ac+H3K27me3+H3K79me1+H3K9me3	0.928494	0.701889
H3K18ac+H3K27me3+H3K4me3+H3K79me1	0.92845	0.708629

H2AK5ac+H3K27ac+H3K9ac+H3K9me3	0.928436	0.696407
H3K27ac+H3K4me1+H3K9ac+H3K9me3	0.928411	0.686931
H3K27ac+H3K27me3+H3K79me1+H3K79me2	0.928352	0.684153
H3K27me3+H3K4me2+H4K5ac+H4K8ac	0.928325	0.69335
H3K27ac+H3K4me1+H3K4me3+H3K9ac	0.928294	0.689874
H3K27me3+H3K4me3+H3K79me1+H4K8ac	0.928101	0.703252
H3K27ac+H3K4me1+H3K9ac+H4K8ac	0.928059	0.682859
H2BK12ac+H3K27ac+H3K4me1+H3K9ac	0.927944	0.694812
H3K23ac+H3K27me3+H3K79me1+H3K79me2	0.927925	0.685082
H3K18ac+H3K27ac+H3K27me3+H4K8ac	0.92792	0.688897
H3K27ac+H3K27me3+H3K56ac+H3K79me1	0.927912	0.696505
H3K27ac+H3K27me3+H3K56ac+H4K8ac	0.927906	0.698417
H2BK15ac+H3K27ac+H3K27me3+H3K4me3	0.927895	0.69314
H3K27ac+H3K4me1+H3K79me1+H3K9ac	0.927867	0.690399
H2AK5ac+H3K27ac+H3K27me3+H3K79me1	0.927845	0.694666
H2AK5ac+H3K27ac+H3K27me3+H4K8ac	0.927834	0.685701
H2BK12ac+H3K27me3+H3K79me1+H3K9ac	0.927813	0.694665
H3K27me3+H3K4me3+H3K79me1+H3K9ac	0.927763	0.687251
H2AK5ac+H3K27ac+H3K4me1+H3K9ac	0.927752	0.695254
H3K27me3+H3K4me1+H3K79me1+H4K8ac	0.927705	0.710103
H3K27me3+H3K79me1+H4K8ac+H4K91ac	0.927682	0.685149
H3K27me3+H3K4me3+H3K79me1+H4K5ac	0.927681	0.694319
H3K27ac+H3K27me3+H3K9ac+H4K91ac	0.927668	0.6826
H2AK5ac+H3K27me3+H3K79me1+H4K8ac	0.927666	0.675337
H3K27ac+H3K4me1+H3K56ac+H4K8ac	0.927661	0.694553
H2AK5ac+H3K27ac+H3K9ac+H4K20me1	0.927548	0.67914
H3K27ac+H3K27me3+H3K4me3+H4K5ac	0.927513	0.706736
H2BK12ac+H3K27ac+H3K27me3+H4K8ac	0.927459	0.690806
H2BK120ac+H3K27ac+H3K27me3+H3K4me3	0.927411	0.687929
H3K27me3+H3K4me1+H3K79me1+H3K9ac	0.927264	0.695756
H3K27me3+H3K79me1+H3K9ac+H4K91ac	0.927263	0.682803
H3K27ac+H3K27me3+H3K4ac+H3K79me1	0.927255	0.694502
H2BK15ac+H3K4me2+H3K9ac+H4K8ac	0.927213	0.68952
H2BK12ac+H3K27ac+H3K27me3+H3K79me1	0.927162	0.696625
H3K27ac+H3K27me3+H3K79me1+H4K20me1	0.92711	0.699796
H3K27ac+H3K27me3+H3K4me1+H3K79me1	0.927096	0.691133
H2AK5ac+H3K27me3+H3K4ac+H3K9ac	0.927089	0.675546
H3K27ac+H3K27me3+H3K4me3+H4K20me1	0.927052	0.692386
H2BK12ac+H3K27me3+H3K4me2+H3K79me1	0.927051	0.679641
H2BK12ac+H3K27me3+H3K79me1+H4K8ac	0.927051	0.697485
H3K27ac+H3K79me1+H3K9me3+H4K8ac	0.927024	0.694588
H3K14ac+H3K27me3+H3K79me1+H3K9ac	0.927008	0.6788
H3K27me3+H3K56ac+H3K79me1+H3K9ac	0.92699	0.694356
H3K27ac+H3K27me3+H3K36me3+H3K4me3	0.926987	0.685538
H2AK5ac+H3K27ac+H3K79me1+H4K8ac	0.926972	0.69254
H3K27me3+H3K79me1+H3K79me2+H4K8ac	0.926962	0.699292
H3K27ac+H3K27me3+H3K4ac+H3K9ac	0.926957	0.682854
H2BK120ac+H3K27ac+H3K27me3+H3K9ac	0.926954	0.700993
H3K27ac+H3K4me1+H3K79me1+H4K8ac	0.926946	0.678918
H3K23ac+H3K27me3+H3K4me1+H3K79me1	0.926946	0.689394
H2AK5ac+H3K27me3+H3K9ac+H4K91ac	0.926937	0.667853

H2BK15ac+H3K27ac+H3K27me3+H3K79me1	0.926927	0.690899
H3K27ac+H3K27me3+H3K4me2+H3K56ac	0.926878	0.699669
H3K27ac+H3K4me2+H3K9me3+H4K8ac	0.926732	0.680161
H2AK5ac+H3K27me3+H3K79me1+H4K5ac	0.926732	0.692423
H2BK20ac+H3K27me3+H3K4me3+H3K79me1	0.926731	0.686929
H2BK20ac+H3K27me3+H3K79me1+H3K9ac	0.926715	0.700774
H2BK20ac+H3K27ac+H3K36me3+H4K8ac	0.926712	0.680506
H2BK15ac+H3K27ac+H3K27me3+H3K4me2	0.92668	0.691365
H3K27me3+H3K4me2+H3K56ac+H3K79me1	0.926639	0.698261
H2BK20ac+H3K27me3+H3K4me2+H3K9ac	0.92662	0.676823
H3K27me3+H3K4me1+H3K79me1+H4K5ac	0.926613	0.680552
H3K27ac+H3K27me3+H3K4ac+H3K4me3	0.926605	0.695099
H3K23ac+H3K27me3+H3K4me2+H3K79me1	0.92658	0.693553
H2AK5ac+H3K27me3+H3K4me2+H3K9ac	0.926562	0.686713
H3K23ac+H3K27ac+H3K27me3+H3K4me2	0.926557	0.701068
H3K27ac+H3K9me3+H4K8ac+H4K91ac	0.926551	0.686003
H3K27ac+H3K27me3+H3K4me2+H4K5ac	0.926499	0.696772
H2BK120ac+H3K27me3+H3K79me1+H4K20me1	0.926492	0.685653
H2BK120ac+H3K27me3+H3K79me1+H3K9ac	0.926491	0.698269
H2BK20ac+H3K27ac+H3K9me3+H4K8ac	0.926476	0.68503
H2BK120ac+H3K27ac+H3K27me3+H3K9me3	0.926442	0.667443
H3K27ac+H3K27me3+H3K4me1+H4K8ac	0.926424	0.697574
H3K18ac+H3K27me3+H3K79me1+H3K9ac	0.926383	0.690359
H3K27me3+H3K4me2+H3K79me1+H4K5ac	0.926378	0.695336
H3K27me3+H3K79me1+H3K9me3+H4K8ac	0.926338	0.696323
H3K27me3+H3K4me2+H3K79me1+H4K8ac	0.926332	0.683303
H3K27ac+H3K27me3+H3K36me3+H3K79me1	0.926331	0.694248
H3K27ac+H3K27me3+H3K4me2+H3K4me3	0.926317	0.698676
H3K27ac+H3K27me3+H3K9ac+H3K9me3	0.926291	0.688608
H2BK20ac+H3K27me3+H3K79me1+H4K8ac	0.92626	0.69587
H2BK120ac+H3K27me3+H3K79me1+H4K5ac	0.926256	0.681392
H3K23ac+H3K27ac+H3K4me1+H3K4me3	0.926239	0.687928
H2BK20ac+H3K27ac+H3K27me3+H3K4me2	0.926227	0.694428
H3K27ac+H3K4me1+H3K56ac+H3K79me1	0.926217	0.693329
H3K18ac+H3K27ac+H3K27me3+H3K4me3	0.926195	0.680226
H3K14ac+H3K27me3+H3K79me1+H4K8ac	0.926175	0.68284
H3K27me3+H3K4me2+H3K79me1+H4K91ac	0.926103	0.70466
H3K14ac+H3K27ac+H3K27me3+H3K9me3	0.926068	0.691423
H2AK5ac+H3K27ac+H3K4me2+H3K9ac	0.926038	0.685063
H3K27ac+H3K27me3+H3K36me3+H3K4me2	0.926016	0.69317
H3K27ac+H3K4ac+H3K9ac+H3K9me3	0.926007	0.682464
H2BK15ac+H2BK20ac+H3K27me3+H3K79me1	0.926006	0.68339
H3K27ac+H3K27me3+H3K79me1+H4K5ac	0.925996	0.680719
H2BK15ac+H3K23ac+H3K27me3+H3K79me1	0.92598	0.691826
H2BK120ac+H3K18ac+H3K27me3+H3K79me1	0.925954	0.674795
H2AK5ac+H3K27ac+H3K9me3+H4K8ac	0.925946	0.694195
H3K27ac+H3K4me1+H3K56ac+H4K5ac	0.925945	0.676377
H2BK120ac+H3K27ac+H3K27me3+H4K8ac	0.925933	0.692068
H3K27ac+H3K27me3+H3K9ac+H4K8ac	0.925925	0.693621
H3K27me3+H3K4me3+H4K5ac+H4K8ac	0.925921	0.699477
H3K18ac+H3K27me3+H3K56ac+H3K79me1	0.925912	0.685189

H2BK15ac+H3K27ac+H3K27me3+H4K8ac	0.925911	0.696544
H3K27ac+H3K4me3+H3K9ac+H4K8ac	0.925902	0.681756
H3K27me3+H3K4me3+H3K79me1+H4K91ac	0.925838	0.696099
H2BK120ac+H3K27me3+H3K4ac+H3K79me1	0.925833	0.687175
H3K14ac+H3K27ac+H3K27me3+H3K4me2	0.925815	0.695373
H3K27ac+H3K27me3+H3K4ac+H3K4me2	0.925796	0.688677
H3K27ac+H3K27me3+H3K56ac+H3K9ac	0.925784	0.682278
H3K27me3+H3K4ac+H3K4me2+H3K79me1	0.925779	0.688394
H3K18ac+H3K27me3+H3K79me1+H3K79me2	0.925756	0.686152
H3K23ac+H3K27me3+H3K79me1+H4K91ac	0.925728	0.681273
H3K27me3+H3K79me1+H3K9ac+H3K9me3	0.92571	0.675607
H3K27me3+H3K79me1+H4K20me1+H4K8ac	0.92564	0.688147
H3K27ac+H3K27me3+H3K4me3+H3K79me2	0.925621	0.678433
H2BK20ac+H3K18ac+H3K27me3+H3K79me1	0.925588	0.679607
H3K23ac+H3K27ac+H3K4me1+H3K9ac	0.925575	0.675906
H3K27me3+H3K79me1+H3K9ac+H4K5ac	0.925572	0.677889
H2BK120ac+H3K18ac+H3K27ac+H3K27me3	0.925557	0.695109
H2BK20ac+H3K27ac+H3K27me3+H3K4me3	0.925555	0.703964
H2BK120ac+H3K23ac+H3K27me3+H3K9ac	0.925545	0.681237
H3K14ac+H3K27ac+H3K27me3+H3K9ac	0.925492	0.688299
H2BK120ac+H3K27me3+H3K4me2+H3K79me1	0.925472	0.694936
H2AK5ac+H3K79me1+H3K9ac+H4K20me1	0.925467	0.679061
H2BK20ac+H3K27ac+H3K4me3+H4K8ac	0.92546	0.68438
H3K27me3+H3K36me3+H3K79me1+H4K5ac	0.925421	0.686836
H3K27ac+H3K27me3+H4K8ac+H4K91ac	0.925418	0.681253
H3K23ac+H3K27me3+H3K79me1+H4K5ac	0.925405	0.679492
H2BK120ac+H3K27ac+H3K9me3+H4K8ac	0.925358	0.683152
H3K27ac+H3K4me2+H3K9ac+H3K9me3	0.925318	0.697102
H2BK15ac+H3K27me3+H3K79me1+H4K91ac	0.925313	0.6824
H2AK5ac+H3K23ac+H3K27ac+H3K27me3	0.925296	0.692367
H3K27ac+H3K27me3+H4K5ac+H4K8ac	0.925262	0.693883
H2AK5ac+H3K27ac+H4K20me1+H4K8ac	0.925259	0.676344
H3K27me3+H3K79me1+H3K9ac+H4K20me1	0.925256	0.681841
H3K27me3+H3K79me1+H3K79me2+H4K5ac	0.925239	0.678837
H3K23ac+H3K27me3+H3K36me3+H3K79me1	0.925233	0.689525
H3K23ac+H3K27me3+H3K4ac+H3K79me1	0.925205	0.688429
H2BK120ac+H2BK12ac+H3K27me3+H3K79me1	0.925198	0.679627
H3K27me3+H3K79me1+H3K9me3+H4K91ac	0.925182	0.687039
H3K27ac+H3K4me1+H3K9ac+H4K20me1	0.925168	0.68368
H3K27ac+H3K4me1+H3K9ac+H4K5ac	0.925166	0.678978
H3K14ac+H3K27me3+H3K79me1+H4K91ac	0.925156	0.690702
H3K23ac+H3K27me3+H3K79me1+H3K9ac	0.925136	0.680056
H2AK5ac+H3K56ac+H3K79me1+H3K9ac	0.925115	0.690911
H2BK120ac+H3K27ac+H3K27me3+H3K4me1	0.925089	0.685426
H2BK12ac+H3K27me3+H3K79me1+H4K91ac	0.924979	0.693712
H3K27ac+H3K27me3+H3K9me3+H4K8ac	0.924978	0.692836
H2AK5ac+H3K27me3+H3K4me2+H4K91ac	0.924948	0.679812
H2BK15ac+H3K27ac+H3K4me3+H4K8ac	0.924948	0.695074
H2BK12ac+H3K27ac+H3K4me3+H4K8ac	0.924918	0.680994
H3K27ac+H3K27me3+H3K9ac+H4K5ac	0.924917	0.683392
H3K27ac+H3K4me3+H3K9ac+H4K20me1	0.924907	0.675206

H2BK120ac+H3K27me3+H3K79me1+H4K91ac	0.924901	0.679942
H3K27ac+H3K27me3+H3K4me2+H3K79me2	0.924895	0.676399
H3K23ac+H3K27ac+H3K4me1+H3K9me3	0.924888	0.686212
H2BK15ac+H3K27me3+H3K4me2+H3K9ac	0.924869	0.684937
H3K27ac+H3K27me3+H3K4me1+H3K9ac	0.92484	0.699157
H2AK5ac+H3K79me1+H3K9ac+H4K91ac	0.924825	0.677279
H3K18ac+H3K23ac+H3K27me3+H3K79me1	0.924816	0.677257
H2BK12ac+H3K27me3+H3K4me3+H3K9ac	0.92481	0.677761
H3K27ac+H3K27me3+H3K4me1+H3K4me3	0.924764	0.690351
H2AK5ac+H3K27ac+H3K4me2+H4K8ac	0.924743	0.689052
H3K27me3+H3K79me1+H4K5ac+H4K8ac	0.924738	0.693542
H2BK120ac+H3K27ac+H3K4me1+H3K9ac	0.924726	0.680699
H2BK20ac+H3K23ac+H3K27ac+H3K27me3	0.924705	0.695386
H3K27me3+H3K79me1+H4K5ac+H4K91ac	0.924705	0.680923
H2BK20ac+H3K27ac+H3K4me1+H4K8ac	0.924703	0.676654
H3K27me3+H3K4me1+H3K4me2+H4K5ac	0.924684	0.676998
H2BK12ac+H3K27ac+H3K27me3+H3K4me2	0.924645	0.687248
H3K14ac+H3K27me3+H3K36me3+H3K79me1	0.924635	0.678431
H3K27ac+H3K9ac+H3K9me3+H4K8ac	0.92459	0.685092
H2BK120ac+H2BK20ac+H3K27me3+H3K79me1	0.924563	0.682763
H3K27ac+H3K4me2+H3K56ac+H4K8ac	0.92454	0.678818
H3K14ac+H3K18ac+H3K27me3+H3K79me1	0.924533	0.675876
H3K18ac+H3K27me3+H3K79me1+H4K5ac	0.924533	0.680992
H2BK12ac+H3K23ac+H3K27ac+H3K27me3	0.924485	0.676275
H2AK5ac+H2BK12ac+H3K27ac+H3K79me1	0.924481	0.692679
H3K18ac+H3K27me3+H3K4me3+H3K9ac	0.924476	0.677192
H3K27me3+H3K4me3+H4K5ac+H4K91ac	0.92447	0.677226
H2AK5ac+H3K27ac+H3K27me3+H3K56ac	0.924438	0.689403
H2BK20ac+H3K27me3+H3K79me1+H4K5ac	0.924425	0.676472
H2BK15ac+H3K27ac+H4K20me1+H4K8ac	0.924411	0.676487
H2AK5ac+H3K27ac+H3K4me3+H4K8ac	0.924382	0.678144
H2BK12ac+H3K27me3+H3K56ac+H3K79me1	0.924374	0.676552
H3K27ac+H3K27me3+H3K4me2+H4K20me1	0.924369	0.675147
H3K27ac+H3K27me3+H3K79me2+H4K8ac	0.924359	0.683677
H3K14ac+H3K23ac+H3K27me3+H3K79me1	0.924342	0.686164
H3K23ac+H3K27ac+H3K9me3+H4K8ac	0.924338	0.682002
H2AK5ac+H3K27me3+H3K4me2+H3K79me1	0.92431	0.687094
H3K27ac+H3K4me3+H3K56ac+H4K8ac	0.924307	0.683309
H2BK15ac+H3K27me3+H3K4me3+H4K5ac	0.924303	0.697837
H2AK5ac+H3K14ac+H3K27ac+H3K27me3	0.924301	0.677467
H2AK5ac+H3K27ac+H3K27me3+H3K4ac	0.924298	0.684768
H2AK5ac+H3K23ac+H3K27me3+H3K79me1	0.924297	0.688514
H2BK120ac+H3K18ac+H3K27me3+H3K9ac	0.924291	0.665058
H3K27me3+H3K4ac+H3K4me3+H4K5ac	0.924288	0.686834
H2AK5ac+H2BK20ac+H3K79me1+H3K9ac	0.924251	0.678987
H2AK5ac+H3K27ac+H3K27me3+H3K4me2	0.924228	0.678892
H3K18ac+H3K27me3+H3K4me2+H3K79me1	0.924214	0.685644
H2AK5ac+H3K79me1+H3K9ac+H4K8ac	0.924213	0.680442
H2AK5ac+H3K27ac+H3K79me2+H4K8ac	0.924205	0.696128
H3K27me3+H3K79me1+H3K79me2+H4K91ac	0.924204	0.675326
H3K27ac+H3K4me2+H4K8ac+H4K91ac	0.924187	0.68228

H2BK20ac+H3K14ac+H3K27me3+H3K79me1	0.924187	0.681766
H2BK120ac+H3K23ac+H3K27me3+H3K79me1	0.924187	0.684581
H3K27ac+H3K4me3+H3K9ac+H3K9me3	0.924151	0.695053
H3K27ac+H3K27me3+H3K56ac+H4K5ac	0.924121	0.696094
H3K18ac+H3K27ac+H3K4me1+H3K56ac	0.924069	0.68087
H3K14ac+H3K27ac+H3K4me1+H3K79me1	0.924061	0.681809
H3K27ac+H3K27me3+H3K4ac+H3K4me1	0.924053	0.686385
H3K27ac+H3K4me1+H3K79me2+H3K9ac	0.924051	0.674
H3K18ac+H3K27me3+H3K79me1+H3K9me3	0.924045	0.68158
H3K27ac+H3K4ac+H3K4me1+H3K79me1	0.924037	0.683438
H2BK120ac+H3K27me3+H3K36me3+H3K79me1	0.924022	0.681344
H3K27me3+H3K4ac+H3K4me3+H3K79me1	0.924009	0.687167
H2BK12ac+H3K27me3+H3K36me3+H3K79me1	0.923996	0.683484
H3K27ac+H3K4me1+H3K56ac+H4K91ac	0.923996	0.677589
H3K27ac+H3K79me2+H3K9me3+H4K8ac	0.923996	0.695645
H2AK5ac+H2BK20ac+H3K27me3+H3K79me1	0.923994	0.682015
H2AK5ac+H3K27me3+H3K4me2+H4K5ac	0.92399	0.691988
H3K27me3+H3K4me2+H3K79me1+H4K20me1	0.923976	0.682227
H3K27ac+H3K4me2+H3K9ac+H4K8ac	0.923974	0.680082
H2AK5ac+H3K27me3+H3K79me1+H4K91ac	0.923959	0.69229
H3K27ac+H3K4me3+H3K9me3+H4K8ac	0.923933	0.686083
H3K14ac+H3K27ac+H3K9ac+H3K9me3	0.923931	0.677164
H3K27me3+H3K4me2+H3K79me2+H4K5ac	0.92392	0.67956
H3K27me3+H3K4me3+H4K20me1+H4K5ac	0.923889	0.69529
H3K27ac+H3K36me3+H3K4me2+H4K8ac	0.923884	0.680753
H3K23ac+H3K27ac+H3K4me3+H4K8ac	0.923879	0.677165
H3K27me3+H3K36me3+H3K79me1+H4K91ac	0.923878	0.684274
H3K27me3+H3K4me3+H3K9ac+H4K5ac	0.923857	0.677908
H2BK20ac+H3K4me2+H3K9ac+H4K8ac	0.92385	0.674924
H3K18ac+H3K27me3+H3K4me2+H4K8ac	0.923846	0.683711
H2BK15ac+H3K79me1+H3K9ac+H4K8ac	0.923833	0.679789
H2AK5ac+H3K27ac+H3K4me1+H4K8ac	0.923829	0.679037
H2AK5ac+H3K27me3+H3K4me3+H3K9ac	0.923828	0.681829
H2AK5ac+H3K79me1+H3K9ac+H4K5ac	0.923811	0.67404
H3K27ac+H3K4ac+H3K4me1+H4K8ac	0.92381	0.677755
H2BK20ac+H3K27me3+H3K4me2+H3K79me1	0.923783	0.685743
H2BK120ac+H3K14ac+H3K27me3+H3K79me1	0.923757	0.679638
H2BK12ac+H3K27ac+H3K79me1+H4K8ac	0.923753	0.685622
H2BK12ac+H3K18ac+H3K27me3+H3K79me1	0.923728	0.686035
H3K27me3+H3K4me2+H3K56ac+H4K5ac	0.923723	0.677629
H3K27ac+H3K9me3+H4K20me1+H4K8ac	0.923716	0.687306
H2BK120ac+H3K27ac+H3K27me3+H3K4me2	0.923709	0.68843
H3K27ac+H3K27me3+H3K36me3+H3K9ac	0.923664	0.674358
H3K18ac+H3K27ac+H3K4me2+H4K8ac	0.923636	0.672931
H3K27ac+H3K4me1+H3K79me1+H4K91ac	0.923634	0.678629
H2BK15ac+H3K27ac+H3K79me1+H4K8ac	0.923631	0.675758
H3K18ac+H3K27ac+H3K27me3+H3K9ac	0.923628	0.687197
H2BK15ac+H3K27ac+H3K4me2+H3K9ac	0.923628	0.692832
H3K23ac+H3K27me3+H3K56ac+H3K79me1	0.923625	0.679511
H2BK12ac+H3K27me3+H3K79me1+H4K5ac	0.923618	0.684991
H2BK120ac+H3K27ac+H3K4me3+H3K9me3	0.923617	0.683991

H2AK5ac+H3K27me3+H3K9ac+H4K8ac	0.923609	0.660478
H3K14ac+H3K27me3+H3K4me2+H4K5ac	0.923594	0.68081
H2BK12ac+H3K27ac+H3K4me2+H3K9ac	0.92357	0.688845
H3K27ac+H3K4me1+H3K4me2+H4K91ac	0.923545	0.681915
H2BK15ac+H3K27ac+H3K9ac+H4K20me1	0.923543	0.673991
H3K14ac+H3K27me3+H3K4me2+H3K9ac	0.923536	0.691677
H3K27ac+H3K4ac+H3K9me3+H4K8ac	0.923516	0.674643
H2BK120ac+H3K27me3+H3K4me3+H3K79me1	0.923515	0.680795
H2BK20ac+H3K23ac+H3K27me3+H3K79me1	0.923508	0.688782
H3K27me3+H3K4ac+H3K4me3+H3K9ac	0.923493	0.678854
H3K27ac+H3K4me1+H3K9me3+H4K8ac	0.923483	0.686097
H3K18ac+H3K27ac+H3K27me3+H3K4me2	0.923478	0.686156
H2BK120ac+H3K27me3+H3K4me3+H4K5ac	0.923475	0.692347
H2BK20ac+H3K27me3+H3K4ac+H3K79me1	0.923473	0.693594
H3K14ac+H3K27ac+H3K4me3+H3K9me3	0.923467	0.678689
H3K27ac+H3K27me3+H3K4me2+H3K9me3	0.92346	0.694206
H3K27ac+H3K4ac+H3K79me1+H4K8ac	0.923404	0.686376
H3K27me3+H3K4me3+H3K9me3+H4K5ac	0.923394	0.681421
H3K27me3+H3K56ac+H3K79me1+H4K91ac	0.923377	0.685533
H3K18ac+H3K27ac+H3K4me1+H4K8ac	0.923293	0.685786
H3K27ac+H3K9ac+H3K9me3+H4K20me1	0.923275	0.675427
H3K27ac+H3K27me3+H3K79me2+H3K9ac	0.923232	0.680861
H2BK12ac+H3K23ac+H3K27me3+H3K79me1	0.923214	0.679947
H2BK120ac+H3K27ac+H3K27me3+H4K5ac	0.923167	0.68383
H3K27ac+H3K4ac+H3K4me1+H3K56ac	0.923162	0.676219
H2BK20ac+H3K27me3+H3K4me3+H3K9ac	0.923161	0.685769
H2BK120ac+H3K27ac+H3K27me3+H3K56ac	0.923129	0.678966
H3K27me3+H3K4me2+H3K4me3+H3K9ac	0.92312	0.677141
H3K23ac+H3K27ac+H3K4me1+H3K4me2	0.923061	0.683625
H2BK120ac+H3K27ac+H3K27me3+H3K36me3	0.923049	0.653178
H2BK120ac+H3K27ac+H3K4me1+H3K79me1	0.923014	0.676966
H3K14ac+H3K27ac+H3K9me3+H4K8ac	0.923001	0.676592
H3K14ac+H3K27me3+H3K4me1+H3K79me1	0.922989	0.676051
H2BK12ac+H2BK15ac+H3K27me3+H3K79me1	0.92294	0.686606
H2AK5ac+H2BK120ac+H3K27me3+H3K79me1	0.922931	0.681271
H3K23ac+H3K27ac+H3K27me3+H3K36me3	0.922915	0.677606
H2BK15ac+H3K27me3+H3K79me1+H4K5ac	0.922896	0.686593
H3K27me3+H3K79me1+H4K20me1+H4K91ac	0.922891	0.6837
H3K27me3+H3K4ac+H3K4me1+H3K79me1	0.922876	0.673333
H3K23ac+H3K27me3+H3K79me1+H4K20me1	0.92287	0.681028
H2BK12ac+H3K27ac+H3K4me3+H3K9ac	0.922868	0.676883
H2AK5ac+H3K27ac+H3K27me3+H4K91ac	0.922803	0.680593
H3K27ac+H3K27me3+H3K4me1+H3K56ac	0.922787	0.686107
H3K27me3+H3K4me1+H3K4me2+H3K79me1	0.922784	0.676709
H3K27me3+H3K4ac+H3K79me1+H3K79me2	0.922783	0.690289
H3K27ac+H3K36me3+H3K4me1+H4K8ac	0.922763	0.67893
H2BK20ac+H3K27me3+H3K79me1+H4K91ac	0.922759	0.696753
H3K23ac+H3K27me3+H3K4me2+H4K5ac	0.922733	0.675712
H3K27ac+H3K27me3+H3K4me1+H4K5ac	0.922722	0.690312
H3K23ac+H3K27ac+H3K27me3+H3K4me1	0.922687	0.686931
H2BK20ac+H3K27ac+H3K4me2+H4K8ac	0.922685	0.676861

H3K27me3+H3K79me1+H3K9me3+H4K5ac	0.922598	0.683882
H3K27ac+H3K4me3+H3K9me3+H4K91ac	0.922537	0.692612
H3K27me3+H3K36me3+H3K4ac+H3K79me1	0.922531	0.687465
H3K27ac+H3K79me1+H4K20me1+H4K8ac	0.922492	0.677385
H3K27me3+H3K4me2+H3K9ac+H3K9me3	0.922479	0.679952
H3K14ac+H3K27ac+H3K4me1+H3K56ac	0.922397	0.67666
H3K27me3+H3K4me2+H3K56ac+H3K9ac	0.922282	0.686501
H3K27ac+H3K4ac+H3K4me2+H4K8ac	0.922239	0.684678
H2BK120ac+H3K27ac+H3K4me1+H3K56ac	0.922184	0.691133
H2BK15ac+H3K23ac+H3K27ac+H3K27me3	0.922184	0.704136
H2AK5ac+H3K14ac+H3K27me3+H3K79me1	0.92215	0.673516
H3K18ac+H3K27ac+H3K9me3+H4K8ac	0.922138	0.69209
H3K23ac+H3K27ac+H3K27me3+H4K20me1	0.922122	0.68037
H2AK5ac+H2BK12ac+H3K27ac+H3K4me3	0.922081	0.679704
H3K27ac+H3K27me3+H3K9me3+H4K91ac	0.922077	0.68399
H3K23ac+H3K27me3+H3K79me1+H3K9me3	0.92205	0.680391
H3K14ac+H3K27ac+H3K79me1+H4K8ac	0.922035	0.683907
H2BK12ac+H3K27ac+H3K4me2+H4K8ac	0.922031	0.681013
H3K14ac+H3K27me3+H3K4ac+H3K79me1	0.922016	0.679052
H3K27me3+H3K4me2+H3K4me3+H4K5ac	0.921998	0.685504
H3K27ac+H3K4me2+H4K20me1+H4K8ac	0.921986	0.70146
H2BK20ac+H3K27me3+H3K56ac+H3K79me1	0.921979	0.693734
H3K27me3+H3K4me2+H3K9ac+H4K20me1	0.921928	0.690496
H3K23ac+H3K27ac+H3K27me3+H3K9me3	0.92191	0.675212
H3K23ac+H3K27ac+H3K4me1+H4K8ac	0.921893	0.685358
H2BK20ac+H3K9ac+H3K9me3+H4K8ac	0.921846	0.684475
H2BK20ac+H3K27me3+H3K79me1+H3K9me3	0.921845	0.676477
H2BK20ac+H3K27me3+H3K36me3+H3K79me1	0.921783	0.676141
H3K27me3+H3K4me3+H3K79me2+H3K9ac	0.921765	0.678182
H3K23ac+H3K27ac+H3K4me2+H4K8ac	0.921754	0.694687
H2AK5ac+H3K27ac+H3K27me3+H3K4me1	0.921739	0.681978
H3K27me3+H3K36me3+H3K4me2+H3K79me1	0.921734	0.682081
H3K27me3+H3K4me2+H3K79me1+H3K79me2	0.921732	0.676064
H3K27ac+H3K9ac+H3K9me3+H4K91ac	0.921698	0.677342
H3K18ac+H3K27ac+H3K9ac+H3K9me3	0.921673	0.67431
H3K18ac+H3K27me3+H3K4me2+H3K9ac	0.921646	0.679143
H3K27me3+H3K56ac+H3K79me1+H3K79me2	0.921645	0.685852
H3K14ac+H3K27ac+H3K4me2+H4K8ac	0.921622	0.675796
H3K27ac+H3K4ac+H3K4me1+H3K9ac	0.921599	0.685135
H3K27ac+H3K4ac+H3K4me2+H3K9ac	0.921591	0.679573
H2BK15ac+H3K27me3+H3K4me3+H3K79me1	0.921553	0.688368
H3K27me3+H3K4ac+H3K79me1+H3K9me3	0.921441	0.684806
H2BK15ac+H3K27me3+H3K4me2+H4K5ac	0.921433	0.693124
H2BK120ac+H3K27me3+H3K56ac+H3K79me1	0.92133	0.675192
H3K27ac+H3K27me3+H3K56ac+H3K79me2	0.921229	0.685052
H3K27ac+H3K27me3+H3K56ac+H4K20me1	0.921194	0.675873
H3K14ac+H3K27ac+H3K27me3+H3K56ac	0.921178	0.680783
H2BK15ac+H3K27ac+H3K4me1+H4K8ac	0.921082	0.678909
H3K27ac+H3K79me1+H4K8ac+H4K91ac	0.92104	0.678929
H2BK12ac+H3K27ac+H3K4me1+H3K79me1	0.921015	0.677651
H2BK20ac+H3K27me3+H3K79me1+H3K79me2	0.920988	0.683195

H2AK5ac+H3K23ac+H3K27ac+H3K4me3	0.920953	0.676639
H2BK15ac+H3K27ac+H3K27me3+H3K56ac	0.920931	0.683447
H3K4me1+H3K4me2+H3K79me1+H3K9ac	0.92086	0.678292
H3K27me3+H3K56ac+H3K79me1+H4K20me1	0.920731	0.67534
H3K27me3+H3K4ac+H3K79me1+H4K20me1	0.92073	0.685334
H2AK5ac+H3K27me3+H3K9ac+H4K20me1	0.920663	0.661387
H2AK5ac+H3K23ac+H3K27me3+H3K9ac	0.920548	0.68604
H2AK5ac+H3K27me3+H3K79me2+H3K9ac	0.920474	0.672856
H2BK12ac+H2BK20ac+H3K27me3+H3K79me1	0.92038	0.678093
H2BK120ac+H2BK12ac+H3K27ac+H3K27me3	0.920364	0.675112
H2AK5ac+H2BK120ac+H3K27me3+H3K9ac	0.919588	0.659763
H2BK120ac+H2BK20ac+H3K27me3+H3K4me3	0.919533	0.656234
H2BK120ac+H2BK15ac+H3K27ac+H3K9me3	0.918976	0.654932
H2BK120ac+H3K23ac+H3K27ac+H3K9me3	0.918593	0.663585
H2BK120ac+H2BK20ac+H3K27me3+H3K4me2	0.917309	0.668771
H2BK15ac+H3K23ac+H3K27ac+H3K9ac	0.916996	0.659032
H2BK120ac+H3K27ac+H3K27me3+H3K4ac	0.916912	0.656529
H2AK5ac+H2BK15ac+H3K27me3+H3K9ac	0.916666	0.658718
H2BK15ac+H3K27ac+H3K56ac+H4K8ac	0.916635	0.66702
H2AK5ac+H2BK120ac+H3K27ac+H3K9ac	0.915949	0.661028
H2BK20ac+H3K27ac+H4K8ac+H4K91ac	0.915204	0.656966
H2AK5ac+H2BK12ac+H3K27ac+H3K9ac	0.914668	0.659302
H2AK5ac+H3K27me3+H3K9ac+H3K9me3	0.914534	0.656281
H3K27ac+H3K9ac+H4K8ac+H4K91ac	0.913763	0.659212
H2BK120ac+H3K14ac+H3K27me3+H4K5ac	0.913683	0.671454
H3K27ac+H3K9ac+H4K5ac+H4K8ac	0.913164	0.659357
H2AK5ac+H3K14ac+H3K27ac+H3K56ac	0.913159	0.672097
H2BK120ac+H2BK15ac+H3K27ac+H4K8ac	0.913045	0.663362
H2AK5ac+H2BK20ac+H3K27ac+H3K27me3	0.912988	0.670462

Table S3: List of the fourteen enhancers used in luciferase reporter assays

eRNA+K27ac+_1	chr1:95113004-95114612
eRNA+K27ac+_2	chr3:55363397-55365168
eRNA+K27ac+_3	chr4:55030444-55032243
eRNA+K27ac+_4	chr5:172204210-172205833
eRNA+K27ac-_1	chr2:106071039-106072921
eRNA+K27ac-_2	chr5:121427975-121429736
eRNA+K27ac-_3	chr5:38782762-38784543
eRNA-K27ac+_1	chr5:73495584-73497044
eRNA-K27ac+_2	chr8:39911413-39912513
eRNA-K27ac+_3	chr6:14881639-14882679
eRNA-K27ac-_1	chr2:15870650-15871999
eRNA-K27ac-_2	chr17:55320202-55321751
eRNA-K27ac-_3	chr12:46008884-46009933
eRNA-K27ac-_4	chr10:36571106-36573012
